# The Strengths and Difficulties Questionnaire (SDQ) in Africa: a scoping review of its application and validation

**DOI:** 10.1186/s13034-017-0212-1

**Published:** 2018-01-11

**Authors:** Nikhat Hoosen, Eugene Lee Davids, Petrus J. de Vries, Maylene Shung-King

**Affiliations:** 1 Adolescent Health Research Unit, Division of Child & Adolescent Psychiatry, University of Cape Town, 46 Sawkins Road, Rondebosch, Cape Town, 7700 South Africa; 2 Health Policy and Systems Division, University of Cape Town, Cape Town, South Africa

## Abstract

**Background:**

Child and adolescent mental health in Africa remains largely neglected. Quick and cost-effective ways for early detection may aid early intervention. The Strengths and Difficulties Questionnaire (SDQ) is globally used to screen for mental health problems, but little is known about its use in Africa. We set out to perform a scoping review to examine existing studies that have used the SDQ in Africa.

**Methods:**

A comprehensive scoping review methodology was used to identify all peer-reviewed studies ever published that have used the SDQ in Africa. Data were extracted and analysed to assess the countries, languages and SDQ versions used, the purpose of the SDQ studies, psychometric properties of the SDQ, and to consider knowledge gaps for future in-country and cross-country studies.

**Results:**

Fifty-four studies from 12 African countries were identified, most from South Africa. Many different languages were used, but authorized SDQs in those languages were not always available on the SDQinfo website. Authors frequently commented on challenges in the translation and backtranslation of mental health terminology in African languages. The SDQ was typically used to investigate internalisation/externalization disorders in different clinical populations, and was most frequently used in the evaluation of children and adolescents affected by HIV/AIDS. Sixteen studies (29.6%) administered the SDQ to participants outside the intended age range, only 4 (7.4%) used triangulation of all versions to generate assessments, and eight studies (14.8%) used only subscales of the SDQ. Only one study conducted thorough psychometric validation of the SDQ, including examination of internal consistency and factor analysis. Where ‘caseness’ was defined in studies, UK cut-off scores were used in all but one of the studies.

**Conclusions:**

The SDQ may be a very useful tool in an African setting, but the scoping review suggested that, where it was used in Africa researchers did not always follow instrument guidelines, and highlighted that very little is known about the psychometric properties of the SDQ in Africa. We recommend comprehensive evaluation of the psychometric properties of the SDQ in various African languages, including internal consistency, factor structure, need for local cut-off values and ensuring cultural equivalence of the instrument.

## Background

Mental health disorders account for at least 14% of the global burden of disease [1], underlining its importance as a global public health concern. Three-quarters of people with mental health problems live in low- and middle-income countries (LMICs) [2, 3], where 75–85% with severe mental health concerns receive little or no treatment [4]. This large treatment gap [5] has multiple underlying causes, including a scarcity in the number of trained mental health professionals [6], lack of advocacy and awareness, as well as the associated stigma of mental illness. When comparing mental health professional availability between high- and LMICs, Europe on average has nine psychiatrists for every 100,000 of the population, while Africa has 0.05 psychiatrists per 100,000 [7]. In addition to the limited mental health services in LMICs, there is also a lack of contextually relevant, rigorous, mental health research in many of these geographical areas [8].

Mental health problems represent the greatest burden of disease among children and adolescents [9]. Ninety percent of children and adolescents live in LMICs, yet only 10% of all child and adolescent mental health (CAMH) research has been conducted in LMICs [8]. In sub-Saharan Africa, projected to be home to 40% of the world’s children by 2025, there are only about 60 qualified child and adolescent psychiatrists and very limited CAMH services. Given the rising incidence of mental health disorders and concomitant resource deficiencies, the treatment gap in Africa is widening [10, 11].

Identification, evaluation and implementation of simple, short and freely-available screening tools for CAMH difficulties may offer a powerful strategy to close the treatment gap, as it may enable the identification of children and adolescents in need of next-step evaluation and treatment. Screening tools might be particularly useful in primary care [12] and in educational settings. Currently a number of screening tools, including the Child Behaviour Checklist [13], SNAP-IV [14], Conners’ ADHD Rating Scales [15] and Social Communication Questionnaire [16] are used in Africa, but it is not clear where or how consistently these tools are used, or to what extent these have been evaluated for their reliability, validity and cultural appropriateness for the diverse populations and contexts in Africa [17].

In CAMH settings around the globe, the Strengths and Difficulties Questionnaire (SDQ) is widely used as a behavioural screening tool, as it has several advantages. It is relatively short, allows for rapid administration, measures both mental health difficulties and competencies, and can be administered by a non-professional with minimal training [18, 19]. The SDQ was developed by Goodman and colleagues [20] in the UK as an open-access, downloadable screening tool, available as a self-report (SDQ-S), parent/caregiver (SDQ-P) and teacher report version (SDQ-T). Goodman [18] recommended the optimal use of the instrument to be a multi-informant tool, with triangulation between parent, teacher and (where appropriate) adolescent self-report. Authorized translations of the tool are available on the SDQ website (http://www.sdqinfo.org) in 83 languages. A strict process of translation, back-translation and authorisation of the tools is maintained by the authors. These processes were designed to ensure the availability of the SDQ in a number of languages whilst maintaining the integrity of the instrument.

The SDQ consists of 25 items to assess a range of ‘strengths’ and ‘difficulties’ as behavioural markers of potential mental health problems. The items contribute to five subscales of five items each with a minimum score of 0 (lowest score) to 10 (highest score): conduct problems, hyperactivity/inattention, emotional symptoms, peer problems, and prosocial behaviour. The sum of the first four subscales generates a total difficulties score, which can range from 0 to 40. From the total difficulties and subscale scores, cut-off scores for clinical ‘caseness’ can be generated. The top 10% of scores based on UK population norms were used to define the ‘abnormal’ range, the next 10% as the ‘borderline’ range, and the remaining 80% of scores as the ‘normal range’ [20]. A higher total difficulty score indicates a greater likelihood of significant problems. Four of the five subscales are scored in a similar way with higher scores indicating more difficulties. The prosocial subscale provides a reverse score where higher scores indicate more prosocial behaviours or strengths.

The reliability and validity of the SDQ has been examined in a number of studies across Europe [20], Asia, Australia and South America [21, 22], but with little or no reference to the use of the SDQ on the African continent. Given the growing awareness of the CAMH needs in Africa [11], it seemed timely to establish the landscape of all research ever performed in Africa that used any versions of the SDQ. We therefore set out to conduct a comprehensive scoping review of the SDQ aimed to describe the use of the SDQ, and to examine the reliability and validity of the SDQ for local use in Africa.

## Methods

The methodological framework for scoping reviews [23, 24] was followed. This included identifying the research question, searching for relevant studies, selecting studies, charting and summarizing the data, and reporting the results. The review objective, inclusion criteria and study methods for this scoping review were specified in advance. Inclusion criteria were (i) any of the versions of the SDQ was used in the study, (ii) the study took place in Africa, (iii) the article was data-driven (i.e. not a review paper), and (iv) the article had been peer-reviewed. We also expressly included studies performed in any language and with no time limit since the development of the SDQ. Given that this was a comprehensive scoping review and not a systematic review, no articles were excluded on the basis of any quality criteria.

### Search strategy

A literature search to identify studies that used the SDQ to evaluate CAMH in Africa since its development in 1997, was conducted until December 2016. Online databases Ebscohost (Africa Wide Information, Medline, PsycINFO) and PubMed were searched with no date limitations, or language restrictions for any African study involving use of the SDQ, in December 2016 and a follow-up search was conducted in April 2017. A general search of Google Scholar was also conducted. The databases were searched using the following keywords: ‘Strengths and Difficulties Questionnaire’, ‘SDQ’, ‘Africa’, ‘children’, ‘adolescents’, and ‘mental health’. An additional search was conducted which included the use of ‘reliability’ and ‘validity’. Searches were not restricted to any search date, but included all available published studies. An additional search was conducted using key words ‘Strengths and Difficulties Questionnaire’, ‘SDQ’ combined with ‘Africa’, ‘adolescents’ and ‘mental health’. The terms ‘reliability’ and ‘validity’ were also used in addition to the combination of the above terms used. Additionally, examination of relevant bibliographies provided further references for review. Titles and abstracts were examined using the inclusion criteria, after which full articles were retrieved.

**Fig. 1:**
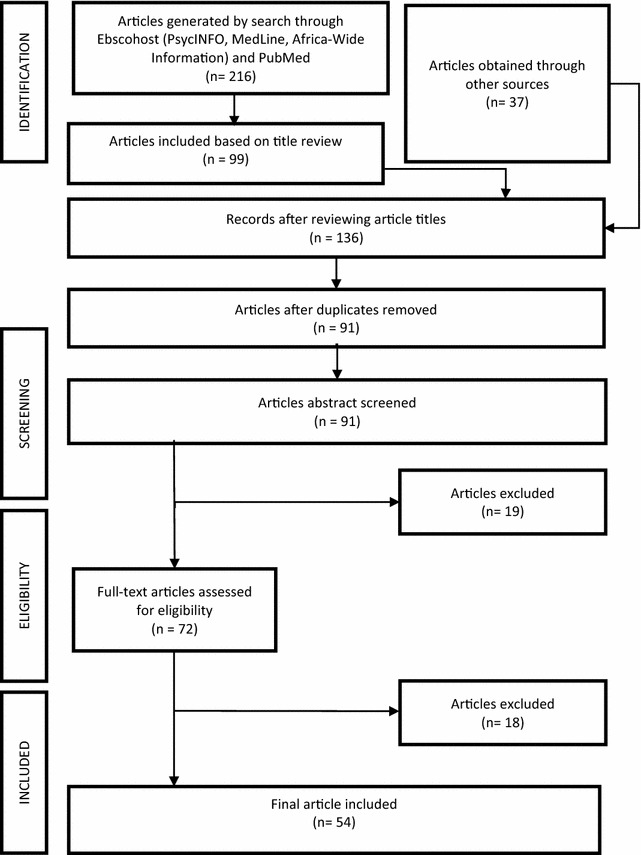
Schematic representation of the literature search process

The initial online search produced 216 articles. Titles were screened for eligibility and 99 articles were identified. A further 37 articles were generated from reference lists and other sources, producing a total of 136 articles. Duplicates were removed, reducing the sample to 91 articles. The abstracts of these were then reviewed to confirm study location and use of the SDQ, producing a total of 72 articles. The 19 articles excluded in the abstract screening phase included review papers, those not conducted in Africa, and articles that used tools with the same acronym as the SDQ (e.g. self-description questionnaire). Eighteen of these were found to be review articles (not apparent from abstract review), reports, one study on African populations living outside of Africa, and presentations using the SDQ in Africa, and were thus excluded. Two independent reviewers assessed the articles for eligibility (NH, ELD). Disagreements were resolved by consensus between the reviewers and, in cases of an impasse, the two senior authors (PJdV, MSK) made the final decision. Figure 1 outlines the process involved in the literature review and final selection of articles. The final sample consisted of 54 articles included in the review (Table 1).

**Table 1:** Strengths and difficulties questionnaire in Africa: data extraction

First author (year)	Location	Age range	Study aim	Theme	SDQ version^a^	Sample	Language	Translation process	Cut-off scores	Differences to UK norms	Alpha scores/internal consistency	Results from use of the SDQ
Kashala (2005) [25]	Kinshasa, Democratic Republic of Congo	7–9	Pilot testing examining stability of the factor structure and reliability of the SDQ	Tool validation	Teacher	1306 learners assessed by teachers	French	None mentioned, French version from SDQ site used	Overall (gender in article) TDS = 19 Emo = 6 Conduct = 5 Hyper = 7 Peer = 5 Pros = 3 Somewhat different to British cut offs	Higher cut-off scores for conduct and low cut-off for Pros, but similar to British cut-offs for Hyper, Emo and Peer	TSD = .81 Emo = .71 Conduct = .64 Hyper = .66 Peer = .35 Pros = .80	Internal consistency satisfactory on all SDQ scales. Using the 90th percentile, cut-off scores were somewhat higher than the published cut-off scores in this younger sample
Cluver (2006) [26]	Cape Town, South Africa	6–19	Examined psychological well-being and mental outcomes of children orphaned by AIDS compared to a matched, non-affected control group	HIV/AIDS, orphans, non-orphans	Self-report (read aloud in Xhosa or English to participants)	60 children living in informal settlements	isiXhosa and English	Translated and blind back-translated	No SDQ norm cut-offs in SA (possibly used percentages to determine three bands)	Using UK cut-off scores high levels of difficulty were found in Peer, Emo and TDS	No mention of alpha scores	Orphaned children and matched controls scored highly for peer problems, emotional problems and total scores. However, orphans were more likely to view themselves as having no good friends, to have marked concentration difficulties (and to report frequent somatic symptoms but were less likely to display anger through loss of temper. Orphans were more likely to have constant nightmares, and 73% scored above the cut-off for PTSD
Kashala (2006) [27]	Kinshasa, Democratic Republic of Congo	7–9	Explore hyperactivity–inattention symptoms and co-existing symptoms of emotional and behavioural problems in African school children and their relationship with health status, socio-demographic factors, and school performance	Hyperactivity–inattention, emotional/behavioural problems, children	Teacher	357 children: 183 were defined as cases due to abnormal scores on the hyperactivity sub-scale, and 174 with normal hyperactivity sub-scale scores	French	None mentioned	Does not specify which cut-off scores were used	Does not specify which cut-off scores were used, nor made a comparison to UK sample	No alpha score reported	Three quarters of hyperactive-inattentive children had co-existing symptoms using SDQ with conduct problems being most common
Cluver (2007) [28]	Cape Town, South Africa	10–19	Investigated psychological consequences of AIDS orphanhood, compared to control groups of children and adolescents orphaned by other causes, and non-orphans	HIV/AIDs, orphans, non-orphans	Peer and conduct problems subscale (self-report)	1025 children and adolescents	isiXhosa	None mentioned	Applied UK cut-off scores of borderline = 4–5, and abnormal = < 6 for peer problems. 14% of AIDS orphans, 9% other-orphans and 8% non-orphans met abnormal criteria. Conduct problems associated with orphanhood by AIDS. Conduct problems in British cut-off for abnormal was 5, and had 5% of AIDS orphans, 3% other-orphans and 4% non-orphan which was lower than what was found in UK levels of conduct problems	Used UK cut-off scores	Did not report alpha scores for two sub-scales used	Controlling for socio-demographic factors AIDS orphaned children were more likely to report symptoms of depression, peer relationship problems, post-traumatic stress, delinquency and conduct problems than both children orphaned by other causes and non-orphaned children Compared to Western norms, AIDS-orphaned children showed higher levels of internalising problems and delinquency, but lower levels of conduct problems
Menon (2007) [29]	Lusaka, Zambia	11–15	Examined emotional and behavioural difficulties in HIV positive Zambian adolescents	HIV, emotional and behavioural difficulties	Self-report; parent	127 children and their	English and Nyanja	Translated and back-translated	Applied UK cut off scores	Zambian sample was more than twice as likely to score outside the normal range to total difficulties, 3 times more likely to have emotional symptoms and 7 times more likely to score in abnormal range for peer problems when compared with UK sample cut offs	Parent form: TDS = .54, Emo = .51, Conduct = .56, Peer = .34, Hyper = .24 Self-report: TDS = .51, Emo = .51, Conduct = .61, Peer = .31 and Hyper = .18	Adolescents who have not disclosed their HIV status were twice as likely to experience high emotional difficulties compared to those who disclosed their HIV status
Okello (2007) [30]	Gulu District, Uganda	11–19	Assessed psychiatric disorders among war-abducted adolescents in northern Uganda, compared to non-abducted adolescents	Psychiatric disorders, war abducted adolescents	Self-report	82 war-abducted and 71 non-abducted adolescents	Language not specified	None mentioned	Applied the following cut offs to the TDS (used in Goodman, 2001): 0–15 = normal, 16-19 = borderline, 20-40 = abnormal	Using Goodman’s (2001) cut offs 51.2% of the war-abducted and 18.3% of the non-abducted adolescents had significant clinical distress on the TDS	No alphas reported	War abducted adolescents had poorer emotional and behavioural adjustment as indicated by their total difficulties scores than non-war abducted adolescents
Cluver, Gardner, and Operario (2008) [31]	Cape Town, South Africa	10–19	Explore mediating effects of stigma and other factors operating on a community level, on associations between AIDS orphanhood and mental health, and associations of four risk factors that can potentially be addressed at a community level (bullying, stigma, community violence, and lack of positive activities) with psychological problems and orphanhood status	HIV/AIDs, orphanhood, stigma, community risk factors, psychological problems	Self-report peer and conduct problems subscale	1025 children and adolescents	isiXhosa	None mentioned	Did not applied any cut-offs	Has not used UK cut-off scores to make comparisons	No alpha scores reported for the two sub-scales used	AIDS orphanhood was significantly associated with higher peer relationship problems AIDS orphanhood was significantly related to higher conduct problems but this association was eliminated when stigma was accounted
Elhamid, Howe, Reading (2009) [32]	Minia, Egypt	6–12	To conduct a population prevalence study of emotional and behavioural disorders among children in this region	Emotional and behavioural disorders	Teacher and parents	1177 children	Arabic	None undertaken. Authors reference that the Arabic version has been previously validated	Applied UK cut-off scores	Applied UK cut-off scores	No alpha scores reported	The prevalence of reported behaviour problems by teachers and parents were much higher in Egypt than in UK
Cluver, Gardner, Operario (2009) [77]	Cape Town, South Africa	10–19	Examine associations between orphanhood, poverty, and psychological distress	HIV/AIDS, orphanhood, poverty, psychological problems	Peer and conduct problems subscale (assumed self-report)	1025 children and adolescents: 425 AIDS orphaned, 241 non-AIDS orphans, 278 non-orphans	isiXhosa	None mentioned	Did not applied any cut-off scores, used continuous scores	Has not used UK cut-off scores to make comparisons	No alpha scores reported for the two sub-scales used	Orphan hood by AIDS was significantly related to peer problems
Doku (2009) [34]	Manya Krobo District, Ghana	10–19	Examine impact of parental HIV/AIDS status and death on child mental health	HIV/AIDS	Self-report	200 children	English	Not applicable/none mentioned	Applied UK cut-offs did not evaluate validity of SDQ	Applied UK SDQ cut-off scores to Ghanaian sample	Did not report alpha scores	Children whose parents died of AIDS showed very high levels of peer problems whilst both orphaned groups scored similarly high on conduct problems Hyperactivity showed no difference and was very low in the entire sample Emotional problems were very high in all the groups except among the non-orphaned children
Menon (2009) [35]	Lusaka, Zambia	11–15	Assess mental health of HIV positive Zambian adolescents in comparison to a school sample and an age matched British normative sample	HIV	Self-report	419 school learners in grades 5-9, 93 HIV positive clinical sample	English and translated version	None mentioned	Has not applied any cut-offs	Has not used UK cut-offs to make comparisons	Did not report alpha scores	Zambian HIV positive adolescents scored higher emotional symptoms and peer problems when compared to a British community sample
Doku (2010) [37]	Ghana	10–18	Assess psychosocial adjustment of children affected by HIV/AIDS	HIV/AIDS, psychosocial	Self-report	50 AIDS orphans, 51 orphans due to other causes, 48 children living with HIV-infected parents, 51 children who did not have HIV/AIDS related deaths in family	English	Not applicable/None mentioned	Did not apply any cut-off scores	Has not used UK cut-off scores to make comparisons	Did not report alpha scores	Total difficulties fort children whose parents died of AIDS but not children whose parents died of causes other than AIDS and children whose parents are infected with HIV/AIDS, scored significantly higher than non-orphaned children whose parents are not known to be infected with HIV/AIDS
Bakare (2010) [36]	Nigeria	4–18	Assess prevalence and pattern of behavioural problems using the teacher reported SDQ among Nigerian children with intellectual disability, to associate behavioural problems with child socio-demographic variables	Behavioural problems	Teacher	44 children with intellectual disability	Language not specified	None mentioned	Use UK cut-off scores	47.7% were classified as borderline and abnormal for total difficulties based on TDS cut off of 12-40	Overall alpha reported = .63	47.7% of children were classified as having behavioural problems in the borderline and abnormal categories on total difficulties clinical scale of SDQ using the cut-off point recommended by Goodman Mild intellectual disability (ID) as compared to moderate, severe and profound ID was associated with highest total difficulties mean score Males were more likely to exhibit conduct and hyperactivity behavioral problems compared to the females
Mueller (2011) [38]	Knysna, South Africa	8–18	Evaluated efficacy of the make a difference about art programme	HIV/AIDS	Self-report	297 children	English, Afrikaans and isiXhosa	Translations performed by bilingual translators and back-checked for accuracy by a second bilingual translator	Did not applied any cut-off scores	Has not used UK cut-off scores to make comparisons	Presents other study’s alpha scores but not their own	Community-based art therapy intervention had no effect on emotional and behavioural problems among children affected by HIV
Puffer (2012) [39]	Muhuru Bay, Kenya	10–18	Examine orphan status, mental health, social support, and HIV risk among adolescents in rural Kenya	HIV, orphan status, mental health, social support	Self-report	325 children	Dholuo	Translated and back-translated	Did not applied any cut-off scores	Has not used UK cut-off scores to make comparisons	Overall alpha score reported = .59	Orphans reported poorer mental health, less social support, and fewer material resources They did not differ from non-orphans on HIV risk indicators Longer time since parental death was associated with poorer outcomes Orphan status was significantly associated with emotional problems
Abbo (2013) [40]	Lira, Tororo, Kaberamaido and Gulu, Uganda	3–19	investigated prevalence, comorbidity, and predictors of anxiety disorders in children and adolescents	Anxiety disorders	Self-report and parent	420 households with children and adolescents	Translated into 4 dialects (not named)	Authors attempted to ensure semantic equivalence between English and local dialects. Forward and back-translation was undertaken for each dialect two teams of mental health professionals were created. The first team translated tools into the local dialect, the second team, blind to the English version, translated the local dialect version into English. A consensus meeting with the two teams was held to resolve any major differences by discussion	Assessed total difficulties score where a score of 16 and above indicated psychological distress	Used a cut-off of above 16 for TDS as an indication of ‘caseness’	No alpha scores reported	Prevalence of anxiety disorders among children and adolescents were higher when having emotional and behavioural problems when measured using SDQ and when having an abnormal or borderline score on emotional symptoms scale
Cortina (2013) [42]	Mpumalanga, South Africa	10–12	Examine prevalence of psychological problems in children, as well as possible risk and protective factors	psychological functioning	Teacher and self-report	1025 children in the 4th and 6th grade	English and Shangaan	Translation, back-translation and adjustment to ensure linguistic equivalence. Two members of the research team native to the area and fluent in Shangaan, one with a Master’s degree in mental health and the other a research officer, translated the questionnaires into Shangaan. Each item was discussed in detail to determine an appropriate translation. A 3rd member of the team also fluent in Shangaan back-translated the items	Did not specify which cut-off scores were used	Teachers ratings found 40% of children as having significant difficulties compared to 10% in UK samples and 17% had reported difficulties in prosocial behaviour compared to 13% in UK samples	TDS = .75, Pros = .80	Teachers reported high levels of behavioural, emotional, conduct and attention problems, with the TDS score from the teacher report SDQ showing that over 40% of children had significant difficulties Poorer psychological outcomes were associated with socio demographic factors like living with a single mother; lower maternal education level; and being from a former refugee family (identified a risk factor)
Atilola (2013) [41]	Nigeria, India, Serbia, Turkey and Indonesia	13–19	Evaluated the prevalence, pattern, and sociodemographic correlates of self-reported mental health problems among adolescents from five countries, to inform the methodology and design of a larger scale study	Mental health	Self-report	1894 adolescents from Nigeria, India, Serbia, Turkey and Indonesia	Not specified	Authors mention that culturally adapted versions of the SDQ for each language and culture in the participating countries were obtained from the SDQ website, “among other sources”. No further explanation of the latter statement	Used UK cut-off scores 4	Applied standard cut offs as recommended in Goodman (1997)	Did not report alpha scores	The prevalence of self-reported mental health problems was 10.5% (range 5.8–15) with conduct and emotional problems being the most prevalent
Kinyanda (2013) [43]	Lira, Gulu, Tororo, and Kaberamaido in Uganda	3–19	Examined prevalence and risk factors of depression in childhood and adolescence in a community sample derived from four disadvantaged districts in north-eastern Uganda	Psychosocial, depression	Self-report, parent	1587 children	Translated into four dialects, but dialects not named	Forward and back-translation was undertaken For each of the four main dialects Two teams of mental health professionals were created. The first team translated tools into the local dialect and the second team blind to the initial English version translated the local dialect version into English. A consensus meeting with the two teams to resolve major differences by discussions was held	Assessed total difficulties score where a score of 16 and above indicated psychological distress	Used a cut-off of above 16 for TDS as an indication of ‘caseness’	No alpha scores reported	Psychiatric co-morbidities/psychiatric problems of emotional distress (assessed by the SDQ), were independently significantly associated with depressive disorder syndromes
Marais (2013) [44]	Mangaung, South Africa	7–11	Investigated the relationship between housing conditions and the socio-emotional health of orphans and vulnerable children in South Africa	OVC	Self-report, parent and teacher	466 orphans, 143 other vulnerable children (non-orphaned)	Sesotho	Following guidelines, the SDQ was translated independently into Sesotho by 2 Sesotho native speakers, who agreed upon a version, which a third native speaker back-translated into English. All translators met and agreed on the final version	Applied UK cut-offs	Applied UK cut-offs	No alphas reported	Orphaned and vulnerable children (OVC) who used other types of toilet facilities (bucket or none) were less likely to have a clinically diagnosable total difficulty score when compared to those using a flush toilet OVC living in a crowded household were more likely to be clinically diagnosed by teachers with a high total difficulties score
Bhana (2014) [45]	Durban, South Africa	10–13	Evaluate a pilot randomized control trial at 2 clinical sites	HIV/AIDS, adolescent, mental health	Self-report	74 families with a child between 10–14 years of age	isiZulu	Translated into isiZulu using standard procedures for translation and back-translation	Did not apply any cut-off scores	Has not used UK cut-off scores to make comparisons	Inter-item reliability = .42–.54	Pre-adolescents who formed part of a family-based, 10 session, intervention saw improvements in mental health, HIV knowledge and adherence to medication
Devries (2014) [46]	Luwero District, Uganda	11–14	To report the prevalence of corporal punishment on children in Uganda and its effects on child mental health and educational outcomes	Violence, corporal punishment	Self-report	3706 students	Unclear if both English and Luganda versions of SDQ were used	All items translated into Luganda and reviewed by a panel of teachers and “Raising Voices” staff to ensure that they would be appropriate for Ugandan child participants and school staff. Thereafter items were cognitively tested and refined iteratively in a sample of ~ 40 children and 20 school staff members from Kampala primary schools to ensure understanding and that meanings of original items were adequately captured. They then surveyed a larger sample of 697 children and 40 staff from Kampala schools to test distributions of items and to test study procedures	Cut-off scores used were highest decile formed the ‘high difficulties’, the next decile formed ‘medium difficulties’ and the remaining 80% formed the ‘low difficulties’	Has not used UK cut-off scores to make comparisons	TDS = .70	For girls exposure to physical violence in the past week was associated with higher levels of poor mental health using the SDQ
Escueta (2014) [47]	Ethiopia, Kenya, Tanzania, India, Cambodia	6–12	Examined relationship between psychosocial well-being and cognitive development orphans and abandoned children (OAC) relative to non-OAC in 5 LMICs to understand factors associated with success in learning	Orphans, psychosocial well-being, cognitive development	Self-report, parent	1480 orphaned and abandoned children	Not specified	None mentioned	Did not apply any cut-off scores	Has not used UK cut-off scores to make comparisons	Overall alpha score reported = .73	An increase in emotional difficulties was found to be associated with delays in cognitive development
Lachman (2014) [48]	KwaZulu-Natal, South Africa	10–17	Examined the relationship between HIV/AIDS and positive parenting	HIV/AIDS, parenting	Parent	685 parent–child dyads with AIDS-ill caregiver and 184 caregiver-child dyad orphaned by AIDS	isiZulu	Translated into isiZulu and back-translated into English	Did not apply any cut-off scores	Has not used UK cut-off scores to make comparisons	TDS = .71	Families who had caregivers who had AIDS or AIDS-orphaned children were significantly related to less positive parenting, higher poverty, higher depression and higher child total difficulties when compared to non-affected families
Marais (2014) [49]	Mangaung, South Africa	7–11	Assess community-based responses to well-being of OVC, compared with their actual mental health to evaluate South African government’s funding approach to CBOs supporting and caring for OVC	OVC, mental health	Self-report, parent, and teacher	465 orphans and 142 other vulnerable children	Sesotho	Adapted and translated in accordance with published guidelines for the translation of instruments in cross-cultural research	Made use of cut-offs to present % of 3 groupings of total difficulties	Does not specify which cut-off scores were used	TDS = .72	Orphaned and vulnerable children with access to medical care reported lower total difficulties than those who had no access to medical care The more a household used its expenditure on food the higher the total difficulties
Skeen (2014) [51]	Malawi	4–13	Explore use of developmental screening tools to measure outcomes of children affected by HIV/AIDS attending community-based organisations (CBO) and to determine what types of CBO provision received by these children	HIV/AIDS, community based organizations	Carers completed a short 10- item version of the SDQ	979 children from South Africa (824) and Malawi (155) as well as 979 caregivers/parents.	Language not specified	None mentioned	Total difficulties score ranged from 0 to 20, higher scores indicating greater behavioural and emotional pathology	Has not used UK cut-off scores to make comparisons	No alpha scores reported	Being a younger child was associated with emotional/behavioural difficulties. Boys were more likely to have higher emotional/behavioural problem scores than girls in both South Africa and Malawi
Sharp (2014) [50]	Free State, South Africa	7–11	To evaluate the construct validity of the caregiver, teacher, and self-report versions of the SDQ	Tool validation	Self-report, teacher, and parent	466 orphans	Sesotho	Adapted and translated in accordance with published guidelines for translation of instruments in cross-cultural research	Parent-form: TDS = 13.5 Emo = 4.5/5.5 Conduct = 3.5 Hyper = 4.5 Peer = not used in this paper Pros = not used in this paper Self-report: TDS = 11.5 Emo = 4.5 Conduct = 1.5 Hyper = 2.5 Peer = not used in this paper Pros = not used in this paper	UK cut-offs: parent-form: TDS = 17 Emo = 5 Conduct = 4 Hyper = 7 Self-report: TDS = 20 Emo = 7 Conduct = 5 Hyper = 7 Teacher-form TDS = 16 Emo = 6 Conduct = 4 Hyper = 7	Alphas: parent-form: TDS = .72, Emo = .60, Conduct = .66, Hyper = .30; Self-report: TDS = .62, Emo = .50, Conduct = .34, Hyper = .26; teacher-form: TDS = .84, Emo = .77, Conduct = .70, Hyper = .67	Orphan care-givers reported cut-offs that were in line with UK cut-offs for emotional symptoms and conduct problems, but less for ADHD and total problems The orphans self-reported cut-offs were significantly lower when compared to UK cut-offs, and teacher reported cut-offs were also lower
Waller (2014) [52]	Western Cape and Mpumalanga, South Africa	10–17	Examined significant risk factors for antisocial behaviour and substance use identified in high income countries (e.g., abuse and poverty), and to determine whether they had predictive effects among South African youth	Antisocial behaviour, substance use	Self-report: conduct problem subscale used only	3515 children	isiXhosa, isiZulu, Swati, Sotho and Shangaan	Translated and back-translated into the five languages	Did not apply any cut-off scores	Has not used UK cut-off scores to make comparisons	Conduct sub-scale alpha score (included items merged from the Child Behaviour Checklist Youth Self-report) = time 1 = .71; time 2 = .65	4 items from the conduct problems sub-scale was used to assess antisocial behaviour among youth that was associated with substance use over time during Time 1 of the study Experience of abuse and community violence at Time 1 predicted antisocial behaviour at Time 2 Being older and male had a significant effect on the increase in antisocial behaviour
Asante (2015) [53]	Accra, Ghana	8–19	Determine the association between psychological functioning and social and health risk behaviours in Ghanian homeless youth	Homeless youth, psychological functioning	Self-report (interviewer administered due to low literacy)	227 homeless children and adolescents	Twi and Ga	None mentioned	Applied UK cut-offs	Applied UK cut-offs	Overall alpha score reported = .72	Only 12.5% of the participants were not exhibiting any psychological symptoms emotional problems were reported by 68.9%, conduct problems by 73.8%, hyperactivity/inattention problems by 53.9% and 88.6% reported peer relationship problems among the homeless youth
Casale (2015) [33]	KwaZulu-Natal, South Africa	10–17	Examined role of caregiver social support as a protective factor for adolescent emotional and behavioural problems	HIV/AIDS, caregiver support	Parent version	2477 adolescent-caregiver dyads	isiZulu	Translated into isiZulu and back-translated into English	Did not apply any cut-off scores	Has not used UK cut-off scores to make comparisons	TDS = .71; Pros = .69	Higher caregiver education is a socio-demographic variable associated with fewer adolescent emotional and behavioral problems for all four TDS subscales Adolescent children who were female and/or orphaned had more emotional problems, while adolescents with older caregivers had fewer conduct problems Caregivers living in the urban sites reported more adolescent child peer and conduct problems, and less prosocial behaviour Lower household socio-economic status was also associated with more adolescent peer problems and less prosocial behaviour
Hermenau (2015) [55]	Tanzania	6–15	Investigate orphans’ experiences of maltreatment and stigmatization to identify factors that relate to their psychological distress	Psychological distress, maltreatment stigmatization orphans	Self-report	89 orphaned and 89 non-orphaned children	Swahili	Translated into Swahili and back-translated into English, using established international guidelines	Did not apply any cut-off scores	Has not used UK cut-off scores to make comparisons	TDS = .63	The main effect of neglect, abuse, and stigmatization correlated significantly positively with orphans’ internalizing and externalizing problems
Collishaw (2015) [54]	Cape Town, South Africa	10–19	Identify predictors of resilient adaptation at child, family and community levels within a group of AIDS-orphaned children, and to consider their collective influence	HIV/AIDS, orphans	Conduct subscale (self-report)S	1025 orphaned children and adolescents	isiXhosa	Translated, back-translated and piloted	Applied UK cut-off scores of borderline = 4–5, and abnormal = < 6 for conduct	Used UK cut-off scores	Conduct = wave 1 = .32, wave 2 = .47; Peer only used in wave 1 = .47	Resilient adaptation is associated with influences across the child, family and community level
Mazzucato (2015) [56]	Angola, Ghana, Nigeria	11–21	To analyse survey data on the psychological well-being of school children and young adults living in transnational families by comparing them with those living with their parents in their countries of origin	Psychological well-being, parent migration	Self-report	2760 students from Ghana, 2243 students from Angola and 2168 students from Nigeria	English and Portuguese	None mentioned	Did not specify which cut-off scores were used	Does not specify which cut-offs were used	No alphas score reported	Children from transnational families (at least one member of the nuclear family lives in a different country) have higher levels of psychological distress than children who live with their parents
Pappin (2015) [57]	Mangaung, South Africa	7–11	Investigated the relationship between socio-economic status and emotional well-being and mental health of orphans	Emotional well-being, orphans	Self-report, parent, and teacher	500 orphans	Not specified	None mentioned	TDS cut-offs: Teacher (normal = 0–11, borderline = 12–15, abnormal/clinically diagnosable = 16 and above); Caregiver (normal = 0–13; borderline = 14–16; abnormal/clinically diagnosable = 17 and above)	Applied UK cut-off scores	Teacher form: TDS = .89 Caregiver form: TDS = .72	Having a female caregiver, 2 daily meals were significantly associated with higher care-giver reported total difficulty scores of orphans Four daily meals, households with higher monthly expenditure and access to medical care associated with lower care-giver reported total difficulty scores of orphans Care-giver with secondary education, employed adult in home and being an older child was associated with lower teacher-reported total difficulties scores Living in a household were a family member receives an old age pension was associated with higher teacher-reported total difficulties scores
Profe (2015) [58]	Cape Town, South Africa	12–17	Examine association between mother, father, and closest grandparent involvement with South African adolescents’ mental health and substance use	Substance use, parent involvement, grandparent involvement	Self-report	512 adolescents	English, Afrikaans and isiXhosa	None mentioned	Applied UK cut-offs for TDS and Pros	Applied UK cut-offs	Internalising problems (Emo and Peer) = .66, externalising problems (Conduct and Hyper) = .76, Pros = .66	Mother and father involvement were both significantly negatively associated with adolescents’ internalizing and externalizing problems Closest grandparent involvement was not significantly associated with adolescents’ internalizing or externalizing problems Mother involvement was significantly positively associated with prosocial behaviour Closest grandparent involvement was positively associated with prosocial behaviour
Abdulmalik (2016) [59]	Ibadan, Nigeria	9–14	Assessed effect of interventions on aggressive behaviour among male primary school learners	Aggressive behaviour	Teacher	37 Male students in the ‘primary five’ school level assigned to an intervention and control group	Not specified	None mentioned	No cut-off scores mentioned	Does not mention which cut-off scores were used and no comparison to UK norms	No mention of alpha scores	The intervention group scored higher for conduct scores than the control group, yet the differences were not significant
Bella-Awusah (2016) [60]	Ibadan, Nigeria	14–17	Determine the effects of a CBT programme on depressed adolescents	Depressed adolescents	Self-report: impact supplement of the SDQ	40 Adolescents who scored 18 or more on the Beck Depression Inventory assigned to an intervention and control group	Both English and Yoruba. All chose English version	Iterative back-translation procedure	Impact score of 2 or more suggest difficulties in psycho-social functioning	Did not use UK cut-off scores to make comparisons	Impact = .72	No significant differences between intervention and control group between baseline and post-intervention, however, improvement in psycho-social functioning/impact 16 weeks follow-up post intervention
Bhana (2016) [61]	KwaZulu-Natal, South Africa	9–14	Determine resilience in perinatal HIV positive adolescents in South Africa	Resilience perinatal HIV + adolescents	Parent	177 caregiver-child dyads who were perinatally HIV infected	isiZulu version	None mentioned, isiZulu versions from SDQ site used	No cut-off scores mentioned	Does not mention which cut-off scores were used and no comparison to UK norms	Pros = .56 TDS = .71	Lower total difficulty scores were associated with decreased caregiver depression, lower caregiver communication difficulties, and increased self-esteem in children. Higher prosocial scores were associated with increased caregiver communication, and the use of wishful (resilient) thinking for coping in children
Chirwa-Mwanza (2015) [62]	Lusaka, Zambia	10–16	Explore associations between relational aggression and psychological well-being among perpetrators in schools in Lusaka	Aggression, psychological well-being	Self-report	170 students in grades 6 and 8, 51% were male	Not specified	None mentioned	Not specified	Did not specify cut-off scores and no comparison made to UK norms	Reported previously published study alpha scores	No significant gender differences were found, however a significant positive associations was found between relational aggression and psychological well-being of perpetrators. Perpetrators of relational aggression were found to have higher conduct problems, peer problems and hyperactivity
Cortina (2016) [63]	South Africa	10–12	Examine cognitive interpretations and psychological functioning of children in rural South Africa	Resilience, HIV/AIDS	Prosocial behaviour subscale (self-report) Total difficulties score (teacher version)	1025 students from a rural, socioeconomic disadvantaged area (40% were from former refugee households)	Shangaan	Translated into Shangaan, and back-translated	No cut-off scores mentioned	Does not mention which cut-off scores were used and no comparison to UK norms	Did not provide alpha scores for SDQ sub-scales	Children who had more negative cognitive interpretations had greater reported difficulties and less pro-social behaviour
Dow (2016) [64]	Moshi, Tanzania	12–24	Establish the prevalence and severity of mental health difficulties among HIV-positive adolescents and to examine the associations between mental health difficulties, stigma, ART adherence and CD4 cell count	HIV/AIDS, ART adherence	Self-report	182 HIV-positive adolescents, just over half were female (54%) and most were attending school (75.8%)	Swahili	Translated into Swahili, and back-translated	A score of 17 or greater for TDS indicated mental health difficulties	Did not use UK cut-off scores to make comparisons	Reported previously published studies alpha scores, but not specific to this study	Mental health difficulties were prevalent in HIV-positive adolescents, and were associated with incomplete adherence to ART and stigma
Doku (2016) [65]	Lower Manya Krobo District, Ghana	10–18	Explore prevalence of child labour and the association with psychological well-being	Child labour, Orphaned and vulnerable children (OVC), HIV/AIDS	Self-report Parent	291 children and adolescents, 51% were female	Not specified	None mentioned	Not specified	No comparison to UK norms	Not specified	Psychological symptoms were higher among children and adolescents who were orphaned by AIDS/caregivers were affected by HIV/AIDS. Children affected by HIV/AIDS (OVC) has significantly more domestic chores and care responsibilities. Child labour mediated the association between orphan status and psychological difficulties
Hecker (2016) [66]	Tanzania	6–15	Examine associations between harsh discipline, internalising mental health problems and cognitive functioning (working memory and scholastic performance)	Harsh discipline, cognitive functioning	Peer problem and emotional symptoms subscales (self-report)	409 primary school students, 52% were male	Swahili	Translated into Swahili, and blind back-translation	Peer: a score of 4-5 indicates “enhanced levels” of peer problems, and 6 indicates abnormal levels of peer problems Emo: a score of 6 indicates “enhanced levels” of emotional problems, and a score higher than 6 indicates abnormal levels of emotional symptoms	Did not use UK cut-off scores to make comparisons	Combined alpha score of.67 was presented	A strong relationship was established between harsh discipline and internalising problems, and were associated with lower working memory and scholastic performance
Hensels (2016) [67]	South Africa and Malawi	4–13	Examine the effect of gender on the development of children attending a community-based organisation in high HIV-affected areas and to examine associations in community-based organisation attendance and changes in gender differences	Gender differences	Parent	979 children from high HIV-affected communities	Not specified	None mentioned	Not specified	Cut-off score not specified and made no comparison to UK norms	Not specified	Males experienced more violence, performed worse at school and more behavioural problems were prevalent than for females at baseline. At follow-up, gender differences persisted, but males reported worse quality of life than females and males were found to experience poorer educational outcomes and behaviour problems
Lentoor (2016) [68]	Eastern Cape, South Africa	31.38–92.78 months (caregivers were 18 or older)	Explore associations in primary caregiver depressive symptomology and psychological functioning of children infected with HIV	Parent depression, HIV/AIDS, psychological well-being	Parent	152 caregiver-child dyads, children were HIV-positive, 87 girls and 65 boys	Not specified	None mentioned	Not specified	Cut-off score not specified and made no comparison to UK norms	Overall alpha score of .73	Depressive symptomology in caregivers were associated with poor psychological functioning in their children
Levetan (2016) [69]	Cape Town, South Africa	13.96 (mean age)	Examine differences in maternal grandmother involvement in grandchildren between those who co-reside and those who do not, and to examine associations between co-residence status, grandmother involvement, adolescent internalising and externalising problems as well as prosocial behaviour	Grandmother involvement, adjustment	Self-report	384 mixed race and black African Grade 8 and 9 learners, 58% were females and 27% lived in 3 generation households with grandmother	Not specified	None mentioned	Not specified	Cut-off score not specified and made no comparison to UK norms	Presented alpha scores from previously published studies	No significant differences were established between involvement of co-residing and non-residing grandmothers. Furthermore, greater maternal grandmother involvement was associated with more adolescent prosocial behaviour, and fewer internalising behaviour problems in three generation households
Mazzucato (2017) [70]	Ghana	11–21	Explore whether being in a transnational family is associated with psychological well-being	Transnational families, psychological well-being	Self-report	2760 secondary school students from areas of high out-migration rates	Not specified	None mentioned	Not specified, just indicated that higher number indicate more psychological distress	Did not specify cut-off scores to make comparisons	The alpha scores for the sub-scales ranged from .70–.73	Being in a transnational family was associated with lower levels of psychological well-being, only in families where parents were divorced or separated
Okewole (2016) [71]	Abeokuta, Nigeria	11.6 (mean age – children) 40.4 (mean age – mothers)	Examine the association between maternal depression and child psychopathology	Maternal mental health, child psychopathology	Self-report	100 mother–child dyads attending a child and adolescent neuropsychiatric hospital	Yoruba	None mentioned, Yoruba version from the SDQ site used	Not specified	Did not specify cut-off scores to make comparisons	Not specified	23% of mothers had a diagnosis of major depressive disorder. 25% of children had abnormal total difficulty scores. A diagnosis of major depressive disorder in mothers were associated with poor total difficulty SDQ scores and poor scores on all SDQ sub-scales except emotional problems
Okewole (2015) [78]	Abeokuta, Nigeria	15.4 (mean age)	Establish the relationship between prodromal psychotic symptoms and psychological distress	Prodromal symptoms, mental health	Self-report	508 secondary school students in the 10th–12th grades.	English	Not applicable/None mentioned	Applied UK cut-off scores	No comparison to UK norms were used	Overall Cronbach alpha score = .63	The prevalence of prodromal symptoms was 20.9 and 11.8% scored abnormal scores on the emotional sub-scale, while 6% scored abnormal scores on the conduct problems sub-scale and 6% for both hyperactivity and peer problems sub-scales. Prodromal symptoms was predicted by higher total difficulty scores and difficulty sub-scales
Puffer (2016) [72]	Migori County, Kenya	10–16	Evaluate intervention aimed at improving family relationships, reduce HIV risk and promote mental health	HIV/AIDS, family relationships, mental health	Version not specified	124 families (237 adolescents and 203 caregivers) from four churches	Not specified	None mentioned	Not specified	Did not specify cut-off scores to make comparisons	Not specified	No effects were found for the intervention on secondary outcomes such as parenting, social support and mental health
Skeen (2016) [73]	South Africa and Malawi	4–13	Establish the relationship between violence exposure and mental health among HIV-affected children	HIV/AIDS, violence, mental health	Version not specified	989 children (834 from South Africa and 155 from Malawi) attending community-based organisations	Not specified	None mentioned	Not specified	Did not specify cut-off scores to make comparisons	Not specified	HIV-negative children who lived with a HIV-positive person experienced more violence, and was followed by HIV-positive children. Interpersonal violence in the home and community predicted internalising and externalising behaviour problems. Harsh physical discipline also predicted behavioural problems in children
Sherr (2016) [74]	South Africa and Malawi	4–13	Examine the effects of caregiver and household HIV on child development	HIV/AIDS, child development	Version not specified	808 caregiver – child dyads, compared over having a HIV caregiver, having HIV in the household and no HIV	Not specified	None mentioned	Not specified	Did not specify cut-off scores to make comparisons	Not specified	Many negative child developmental outcomes was associated by HIV burden, and was mediated by caregiver depression levels. The familial burden of HIV at baseline affected child behavioural problem as follow indirectly as a result of depression of the caregiver. Both internalising and externalising behaviour problems were indirectly affected by familial HIV burden and caregiver depression
Thumann (2016) [75]	Luwero District, Uganda	7–18	Examine whether individual and contextual factors in the school environment were associated with mental health difficulties, including the association of mental health difficulties with violence exposure and differences by gender	School environment, violence, mental health	Self-report	3565 primary school students	Not specified	None mentioned	Binary variable created for those above and below the 80th percentile	Did not compare to UK norms but applied rule that those above the 80^th^ percentile would be categorised as “borderline/abnormal” as outlined by Goodman, Meltzer and Bailey (1998)	Overall Cronbach alpha score = .69	Experiences of violence by staff or students were associated with mental health difficulties. Low school connectedness also increased the odds of mental health difficulties. Effect of violence on mental health difficulties were not mediated by school connectedness and there were no significant differences across gender. Attending an urban school increased the odds of mental health difficulties
Tucker (2016) [76]	KwaZulu-Natal, South Africa	10–17 (children) Over 18 (caregiver)	Establish the impact of caregiver ill-health and child orphan status on prosociality in HIV-affected settings	HIV/AIDS, orphans, prosocial behaviour	Prosocial behaviour sub-scale only: self-report and parent versions only	2136 child-caregiver dyads in an HIV-epidemic community	English and isiZulu	None mentioned	Applied UK cut-off scores	Used UK cut-off scores but no comparison to UK norms	Pros (Self-report) = .89 Pros (parent version) = .69	Differences were found in the rating of prosocial behaviour in households where caregivers were ill and children were orphaned due to AIDS. Caregivers reported low prosocial behaviour in children, while the children rated high prosocial behaviour

*TDS* total difficulties score, *Emo* emotional symptoms, *Conduct* conduct problems, *Hyper* hyperactivity/inattention, *Peer* peer problems, *Pros* prosocial behaviour

^a^Self-report, parent or teacher version

### Data extraction

Full PDF versions of all included articles were collated by the first author and analysed by the first and second author to extract relevant information for the review. A summary table was generated (Table 1) which included the following fields: author, year, study location, participant age, study samples, aim of the paper, theme of study, SDQ report version used, SDQ language version used, translation process, clinical cut-off scores used, comparison to UK norms, internal consistency of the SDQ, as well as the results of each study.

### Data analysis

The data gathered and extracted from the articles were analysed using thematic analysis. In keeping with the aim of the review to examine the use and cultural appropriateness of the SDQ in Africa, the deductive themes extracted included specific examination of the location of studies, languages used, instrument translations, cultural comments about use, and psychometric properties of the instrument. Other inductive themes which emerged during extracting and synthesis of data, were research theme of use, versions and subscales used. All findings are summarised in Table 1.

## Results

The review identified 54 studies [25–78], conducted in 12 African countries (Table 1). Below we summarise data based on (i) sociodemographic descriptors, (ii) SDQ versions, (iii) SDQ triangulation, (iv) tool properties and validation, (v) translation, back-translation and authorization, and (vi) the purpose of using the SDQ in Africa.

### Sociodemographic descriptors

**Fig. 2:**
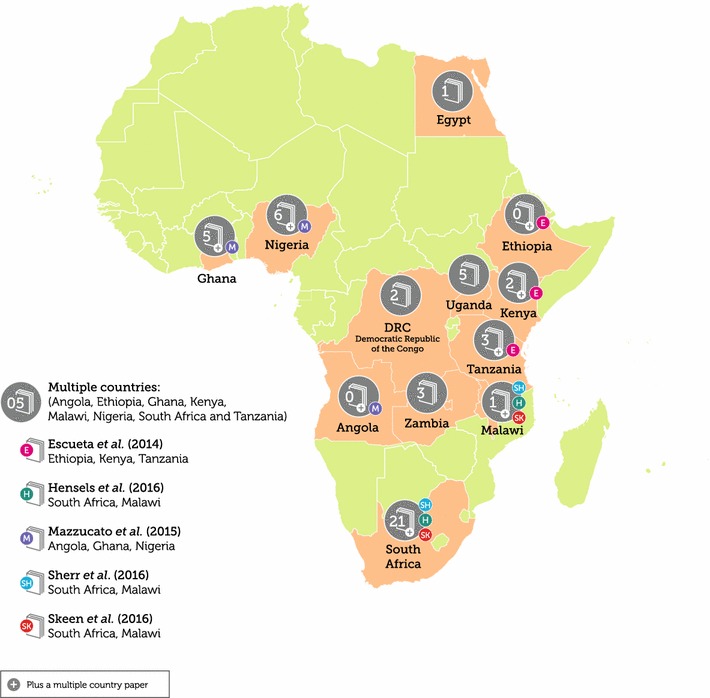
Geographical location of SDQ studies in Africa

The 12 countries represented in the review included South Africa (n = 21 plus an additional 3 from two-country studies between South Africa and Malawi), Nigeria (n = 6 plus an additional study from a three-country study between Nigeria, Angola and Ghana), Ghana (n = 5, plus the additional three-country study with Nigeria and Angola), Uganda (n = 5), Tanzania (n = 3, plus an additional three-country study between Tanzania, Kenya and Ethiopia), Kenya (n = 2, plus the additional three-country study), Zambia (n = 3), Democratic Republic of Congo (n = 2), Angola (n = 1, based on the three-country study with Nigeria and Ghana), Egypt (n = 1), Ethiopia (n = 1, based on the three-country study with Tanzania and Kenya), and Malawi (n = 1, plus the additional 3 two-country studies with South Africa) (Fig. 2). As listed above, five studies represented samples from two countries, namely South Africa and Malawi in three studies, and two three-country studies, namely Tanzania, Kenya and Ethiopia as well as Nigeria, Angola and Ghana. All studies included male and female participants who ranged in age between 31 months and 24 years. Interestingly, 16 studies (29.6%) administered the self-report version to participants as young as six and as old as 24 years, despite the SDQ administration guidelines recommending the self-report version for 11–17 year olds [26, 30, 34, 37–39, 41, 42, 54–56, 62, 64, 66, 70, 75].

### SDQ versions used

**Fig. 3:**
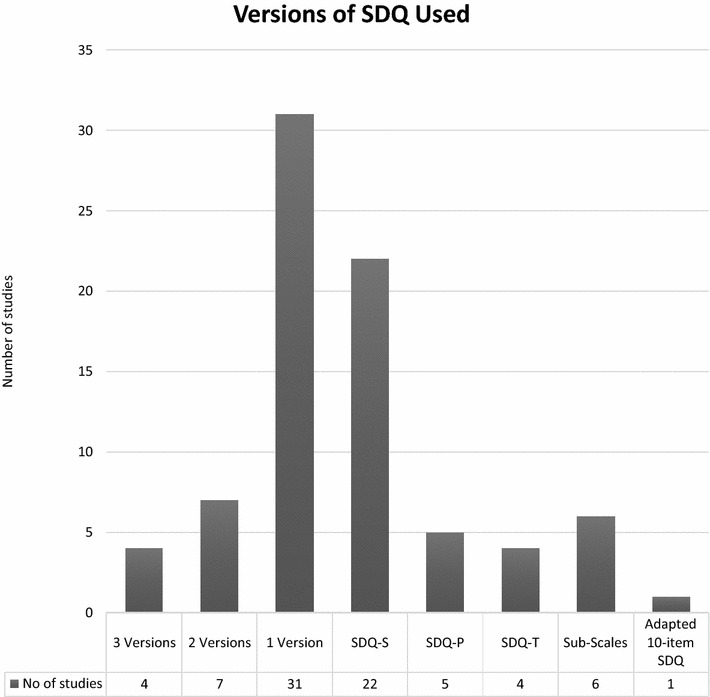
SDQ versions used in studies from Africa

Of the 54 studies, four (7.4%) used all three versions (parent, teacher and self-report) of the SDQ [44, 49, 50, 57] (see Fig. 3). Seven studies (12.9%) employed 2 SDQ versions [29, 32, 40, 42, 43, 47, 65]. Thirty-one studies (57.4%) used only one version of the SDQ, of which 22 used the SDQ-S [26, 30, 34, 35, 37–39, 41, 45, 46, 53, 55, 56, 58, 60, 62, 64, 69–71, 75, 78], four the SDQ-T [25, 27, 36, 59] and five the SDQ-P [33, 48, 61, 67, 68] (see Table 2 for summary of results by SDQ versions). In the remaining 12 articles (22.2%), three used only the peer and conduct problem subscales from the SDQ self-report version [28, 31, 77], one used only the peer and emotional problem subscales [66], while a further two articles used only the conduct subscale of the self-report SDQ [52, 54]. In addition, the self-report prosocial subscale was used together with the parent prosocial subscale [76] and the teacher total difficulty score [63]. Three studies did not specify which version of the SDQ was used [72–74]. One study used an unauthorised modified 10-item version of the SDQ-P [51] which assessed total difficulties. Overall, the SDQ self-report version was most frequently used (32 studies, 56.1%), followed by the parent report (15 studies, 26.3%), and the teacher report version (10 studies, 17.5%).

**Table 2:** Summary of results by SDQ version (SDQ-S, SDQ-P and SDQ-T) used

	SDQ self-report	SDQ parent report	SDQ teacher report
Total number of papers identified	35^a^	18^a^	10^a^
Location	South Africa (n = 9) Ghana (n = 6) Uganda (n = 5) Nigeria (n = 5) Tanzania (n = 3) Zambia (n = 3) Kenya (n = 2) Angola (n = 1) Ethiopia (n = 1)	South Africa (n = 9) Uganda (n = 2) Egypt (n = 1) Ethiopia (n = 1) Ghana (n = 1) Kenya (n = 1) Malawi (n = 1) Tanzania (n = 1) Zambia (n = 1)	South Africa (n = 5) Nigeria (n = 2) Congo (n = 2) Egypt (n = 1)
Translation process			
Translated and back-translated	15	5	1
Translation guide used	4	3	3
No mention of process	8	7	4
Not applicable	4	0	2
Common themes	HIV/AIDS and orphans (n = 12)	HIV/AIDS and orphans (n = 10)	Orphans and emotional and behavioural problems (n = 6)
Psychometric properties			
Included in study	14	9	6
No mention in study	16	6	4
Cronbach alpha score ranges	0.18–0.80	0.24–0.73	0.35–0.89

^a^Number includes multi-country and triangulated studies (which might inflate number of studies)

### SDQ triangulation

Triangulation of SDQ data through the use of multiple informants is recommended by the developers [79]. Only four (7.4%) [44, 49, 50, 57] of the 54 studies in the review made use of all versions (parent, teacher and self-report) of the SDQ to satisfy triangulation of the screening tool. All four studies took place within South Africa, three made use of the Sesotho versions of the SDQ (parent, teacher and self-report), while the other study did not specify the language version used. The remaining 50 studies in the review did not make use of triangulation.

### Psychometric properties and validation

Twenty-six studies (48.1%) included an evaluation of some aspect of the psychometric properties of the SDQ, for instance, 25 (46.2%) reported the Cronbach alpha scores for internal consistency [25, 29, 33, 36, 39, 42, 45–50, 52–55, 57, 58, 60, 61, 66, 68, 70, 71, 75, 76]. Four studies (7.4%) also presented Cronbach alpha scores from previously published studies [38, 62, 64, 69]. The overall reported Cronbach alpha scores ranged from 0.18–0.89. For the SDQ-S Cronbach alpha scores were reported in 14 studies (25.9%) and ranged between 0.18 and 0.80. Sixteen studies (29.6%) did not report on the psychometric properties for the SDQ-S. For the SDQ-P Cronbach alpha scores were reported in 9 studies (16.7%) and ranged between 0.24 and 0.73. Six studies (11.1%) did not report on psychometric properties of the SDQ-P. SDQ-T scores ranged between 0.35 and 0.89 in the 6 studies (11.1%) that reported Cronbach alphas, while four studies (7.4%) did not report on internal consistency or other psychometric properties of the SDQ-T (see summary in Table 2). One study made use of inter-item reliability on the SDQ-S [45] and another generated a composite score for all subscales used on the SDQ-S [52]. Overall, twenty-four studies (44.4%) failed to report or examine Cronbach alpha scores. One study examined the factor structure of the SDQ among children in the Democratic Republic of Congo (DRC) using the SDQ-T, and the same study established clinical cut-off scores on the SDQ-T for the DRC population [25]. One study examined the construct validity of all three versions of the SDQ in a sample of Sesotho children and adolescents in South Africa, as well as their caregivers and teachers [50]. To do this, the SDQ was administered alongside the computerised diagnostic interview schedule for children (4th edition; CDISC-IV) to the sample and the subscales were matched with the CDISC-IV diagnostic groups based on the DSM-IV criteria [50]. The emotional problem subscale was matched to detect anxiety disorders and affective disorders on the CDISC-IV, while the conduct problem subscale was matched to detect oppositional-defiant disorder and conduct disorder. In addition, the hyperactivity/inattention subscale was matched to detect ADHD. The peer problem and prosocial behaviour scales were not matched as there were no criteria in the CDISC-IV to find matched equivalent diagnostic groups [50]. The study [50] suggested support for the SDQ-P version, but not for the SDQ-T and SDQ-S, and provided clinical cut-offs with some caution.

### Translation, back-translation and authorization

With regards to translation and adaptation of the SDQ, 23 studies (42.6%) reported the process of translation and back-translation of the SDQ. Of these, eight translations were not listed as authorized on the SDQ website and have not been translated in consultation with the tool developers, namely Nyanja [29], Dholou [39], Sesotho [44, 49, 50, 52], Luganda [46], Swati [52], Shangaan [52, 63], Twi [53] and Ga [53]. Challenges raised in the process of translations and adaptions included absence of a linguistic equivalent in some of the local languages for words such as ‘fidget’ [80], ‘nervous’ or ‘fidgeting’ [35]. Of the studies in the review, the SDQ was available in seventeen different languages, while twenty studies did not specify language(s) used. Five studies, two in South Africa [26, 54] and one each in Ghana [53], Tanzania [66] and Nigeria [71] reported the use of interviewer support when using the self-report SDQ, due to low literacy levels. There are no authorised procedure at http://www.sdqinfo.com for interviewer administration of the SDQ. Of the 23 studies reporting on the translation and adaption of the SDQ, only seven provided evidence of the evaluation of translated words and its equivalences [35, 39, 40, 43, 49, 64, 66], evidence of translation team discussions regarding semantics was found in one study [46], and another used a qualitative approach to perform a cognitive review of the translated version of the SDQ [50].

In addition, three studies used English versions of the SDQ and thus did not require translation [34, 37, 78]. Of the remaining 51 studies, three used translated versions of the SDQ from the official site [25, 61, 71] and 27 did not discuss any process of translation. Elhamid and colleagues [32] did not discuss translation of the SDQ used but referenced the Arabic version that had been previously validated.

While some authors discussed the process and implications of instrument translation in both their methods and discussion, this was not universal, and showed varying degrees of detail across studies. Seventeen studies mentioned that back-translations were performed. Four studies did not provide specific details about the translation process, but rather referenced that the process was undertaken according to published guidelines or standard procedures [45, 49, 50, 55].

There is a distinct lack of consistent examination and reporting of translation processes for this tool. Devries [46] and Cortina [42] represent good examples of processes that take into account cultural appropriateness and linguistic equivalence in the translations of the SDQ. The use of trained mental health professionals who are native speakers, independent forward and blind back-translations, collaboration with teachers and staff to assess the tool, as well as pilot testing of tools in small and then bigger samples, are all steps that can be taken to assess the SDQ in a setting before use. The translation process for studies that made use of the SDQ-S commonly reported the use of a translation and back-translation process (n = 15; 27.8%), while the studies using the SDQ-P (n = 7; 13.0%) and SDQ-T (n = 4; 7.4%) more frequently did not report on the translation process (see Table 2).

### Purpose of SDQ use in Africa

Use of the SDQ in Africa fell into two broad categories, (i) assessing internalising and externalising problems among children and adolescents in Africa, and (ii) assessing mental health in the context of HIV/AIDS.

#### Internalising and externalising problems among children and adolescents in Africa

The SDQ was used to examine internalising and externalising problems of children and adolescents in Africa in 21 of 54 studies. Some of the internalising disorders included emotional problems [27, 32, 36, 51, 59, 62], anxiety disorders [40], depression [43, 60], psychological functioning and mental health in homeless youth [53], orphans and vulnerable children exposed to maltreatment and stigma [44, 47, 55, 57], war-abducted adolescents [30], psychological well-being as it relates to parental migration [56, 70] and lack of parenting support [30]. Externalising problems examined included hyperactivity/impulsivity [27], behavioural problems [27, 32, 36, 51, 59, 62], effects of corporal punishment on mental health and educational outcomes [46, 75], antisocial behaviour and substance use [52], and caregiver association with substance use and mental health [58].

#### Mental health difficulties in the presence of HIV/AIDS

The majority of articles (24 of 54) identified in this review used the SDQ to explore child and adolescent mental health in the context of HIV/AIDS (17 studies from South Africa, three from Ghana, two from Zambia, Kenya and Malawi, and one from Tanzania) and compared mental health and HIV associations between orphaned and non-orphaned children in South Africa [26, 28, 31, 54, 76, 77]. Peer problems were among the common mental health difficulties for children orphaned due to AIDS [26, 28, 31, 77], followed by posttraumatic stress disorder [26, 28] and conduct problems [28, 31]. Studies included examination of emotional and behavioural difficulties in HIV positive adolescents [29, 61, 64], the impact of parental HIV/AIDS status and death on the mental health of the child [34], psychosocial adjustment of children affected by HIV/AIDS [37], evaluation of community art therapy intervention on the mental health of children affected by HIV [38], randomised controlled trials pilot evaluation [45], caregiver social support [33], positive parenting [48], and in the provision and outcomes of community-based organizations for children and adolescents [54, 67, 73, 74].

## Discussion

Given the divide between need for and access to CAMH services particularly in low- and middle-income settings such as in Africa, screening tools such as the SDQ that are simple, accessible and freely-available has the real potential to improve early identification and access to care in Africa and other LMIC settings. However, for clinically-meaningful implementation, it is essential that any instrument not designed in the context should be examined to ensure it has good psychometric properties (e.g. is reliable and valid in the new context), is culturally appropriate, and is used in adherence to the guidelines of the developers of the instrument. We therefore set out to explore the current knowledge-base about the use of the SDQ in Africa.

We identified 54 peer-reviewed publications from 12 African countries, most from South Africa. The SDQ was typically used to investigate internalisation/externalization disorders in different clinical populations, including vulnerable populations such as orphans, children in war-torn areas, and migrants. Interestingly, the SDQ was most frequently used in the evaluation of children and adolescents affected by HIV/AIDS. Many different languages were used, but authorized SDQs in those languages were not always available on the official SDQinfo website. Authors frequently commented on challenges in the translation and back-translation of mental health terminology in African languages. Sixteen studies (29.6%) administered the SDQ to participants outside the intended age range, only 4 (7.4%) used triangulation of all versions to generate assessments, and 8 studies (14.8%) used only subscales of the SDQ. Where ‘caseness’ was defined in studies, UK cut-off scores were used in all but one of the studies. Only one study conducted a thorough psychometric validation of the SDQ, including examination of internal consistency, generating cut-off scores, and factor analysis [25].

The African continent is highly multi-cultural and multilingual. Screening tools such as the SDQ initially validated for a UK population, have been reported to have good psychometric properties in high-income settings [81], but, as shown in this scoping review, these findings have not been replicated in Africa. The results presented in the current review suggest that the SDQ has been used in several African countries among various groups of children and adolescents, without comprehensive validation for use in these settings. The many studies that have used the SDQ in Africa reporting limited or no validation therefore raises concerns about the robustness of findings reported in the African CAMH literature to date.

We were surprised to observe the application of the SDQ outside the scope and guidelines of the instrument. We strongly believe that all researchers who work in Africa have an ethical duty to ensure adherence to instrument guidelines and to work in collaboration with tool developers. Given the limited research resources available in Africa, we have to ensure high-quality research at all times.

An additional potential area of concern was the limited use of triangulation of measures in only four studies all conducted in South Africa. Whilst the philosophical position of the SDQ developers is to encourage triangulation of data in clinical practice, many different research questions could be answered without triangulation of data, for instance, investigation of the psychometric properties of a specific version of the SDQ [82]. We also acknowledge that the low rate of triangulated use of the SDQ may have been purely pragmatic, for instance, for the assessment of homeless youth, a self-report version may have been the only option for assessment. For this reason, we are cautious not to overinterpret the limited use of triangulation reported here. Further examination of triangulated data would, however, be of interest to examine measurement invariance between parent, teacher and self-report versions, and cross-culturally. To date, measurement invariance for the different versions of the SDQ have yielded conflicting, and ambiguous findings [83, 84].

Context-specific validation of the SDQ is crucial, as demonstrated in a recent South African study that evaluated the psychometric properties of the SDQ in 3451 adolescents aged 12–16 [82]. The results showed reasonable, yet variable, internal consistency, but identified significant gender-based differences in scores. More importantly, it showed a very unusual profile of ‘caseness’. Using UK cut-off values (designed to identify the top 10% of scorers), 26% of 12–16 year olds were found to be at high risk of emotional problems and 33.7% to be at high risk for peer problems (de Vries et al. 2017 [82], Table 3). Based on these observations, the authors raised the need for extreme caution in making cross-country comparisons. For intra-county clinical use, the study recommended the use of local cut-off scores to define clinical ‘caseness’ in any screening procedure. For example, a suggestion had been made to use the SDQ-S for mass screening as part of the South African Integrated School Health programme (personal communication, MSK). UK cut-off scores for caseness would have led to a two- to three-fold overidentification of young people ‘at risk’, which could have caused potential distress among young people, and could have placed the already very limited mental health services under overwhelming strain.

The scoping review and our recent findings [82] also raised the question about the cultural appropriateness and challenges with translation and back-translation of the SDQ to ensure valid use [85]. Africa has 2000–3000 languages, and only a handful of these have been included in SDQ translations. We suggest that it will be important to understand the local perceptions of mental health and the linguistic subtleties in the description of symptomatology, in order to evaluate how best to translate and validate instruments such as the SDQ [82, 86].

Our findings highlighted a few additional important issues. Firstly, there has been a steadily growing body of research on CAMH in Africa, suggesting a heightened awareness amongst practitioners and researchers of the need to identify and document mental health conditions. In this review we identified a significant number of articles in 2016, suggesting a recent growth spurt in research. The review further highlighted that mental health challenges for African children and adolescents often occur within contexts of significant large-scale trauma, such as in situations of conflict, conflict-linked migration and the persistent and long-standing HIV/AIDS epidemic with the accompanying personal and societal devastation. Close to 18 million children and adolescents on the African continent have been orphaned due to HIV/AIDS [87], it is therefore not surprising that 27 studies in the review examined children and adolescents’ mental health in relation to HIV/AIDS. Some studies were set among homeless children and adolescents [88] and those exposed to war [43]. This is not necessarily the case in other regions in which the SDQ had been applied. This raises the empirical question about the extent to which the SDQ, developed in a high-income setting, typically applied in a stable, non-crisis environment could, even if applied with great due diligence, be appropriate for the identification of mental health challenges in such challenging contexts.

### Lessons learnt for future SDQ research in Africa

One of the key findings from our review is the importance of ensuring that use of screening tools such as the SDQ should be used in adherence with developer guidelines and authorization. Where instruments are translated without authorization, used outside the prescribed age-ranges or where only subsections or items are used, there is a real risk of incorrect and potentially misleading findings.

Secondly, given the fact that only one study in the review period had performed a comprehensive evaluation of the psychometric properties of the SDQ in a specific African country, there is a clear need to see similar studies in other African countries. The study by de Vries [82] illustrated the potential value of such evaluation. We support the recommendation that UK cut-offs should not be used to determine ‘caseness’ in African countries and that further validation work is required to compare normative cut-off values with gold standard diagnostic instruments to establish the true sensitivity and specificity of the SDQ [82].

de Vries and colleagues [82] also recommended that the SDQ could be useful as an ‘in-country’ instrument, but that it should be used with great caution as a ‘cross-country’ comparative measurement tool. There is clearly an increasing realisation of the need for cross-cultural measurement instruments in CAMH [83, 84, 89]. However, we acknowledge that, to date, most CAMH screening tools have not been developed with a global user in mind. In an Africa setting this task is significant and a range of challenges will need to be considered from the subtle cultural, ethnic, pragmatics of language [82, 89] to the large number of languages and variability of literacy levels. de Vries [82] recommended qualitative exploration of the cultural use of mental health language in order to develop mental health measurements that can capture similar global concepts appropriately and adequately in local settings.

In terms of policy-making, it is important to emphasize that measurement instruments should be selected for inclusion in policies and implemented not based on evidence and use in high-income countries, but only when local evidence has been generated to support the safe and meaningful use of these tools.

### Limitations

We acknowledge a number of limitations in this study. Only four databases were searched to identify articles for this review. It is therefore theoretically possible that some studies were not identified. However, the four databases included typically covers the significant majority of all peer-reviewed journals. In particular, we did not have any language exclusion in order to ensure that we were able to identify potential publications from French- and other African language-speaking sources. In terms of translation/back-translation and authorization of SDQs, it is possible that there may have been approved SDQ versions not yet included on the SDQinfo website. For instance, the most recent Afrikaans and isiXhosa SDQ-S used in de Vries et al. [82] have been authorised by Goodman but are not yet uploaded on SDQinfo. It is possible that some of the ‘languages’ and dialects used in some of the studies may not have required specific translation/back-translation and authorization.

## Conclusion

The SDQ is an easy to use, reliable screening tool for early identification of mental health disorders in children and adolescents, and has been used in numerous countries and languages. The comprehensive scoping review of the SDQ in Africa showed that it has been used in about a quarter of African countries, and that it may be a useful screening instrument to identify children and adolescents at risk of mental health problems. However, the limited and variable psychometric knowledge about the SDQ in Africa suggests that the tool should be used with caution to define ‘caseness’, and that research teams across the continent should perform careful psychometric evaluation of the SDQ in their countries and languages. We suggest that, apart from standard psychometric evaluation, the multicultural and multilingual nature of Africa also necessitates careful cultural evaluation of the instruments to ensure equivalence for clinical and research use. It throws down the gauntlet for practitioners, researchers and academics who work in this field, to pay meticulously attention to the rigour with which these instruments are applied.
