# Correction to: The Strengths and Difficulties Questionnaire (SDQ) in Africa: a scoping review of its application and validation

**DOI:** 10.1186/s13034-018-0217-4

**Published:** 2018-01-31

**Authors:** Nikhat Hoosen, Eugene Lee Davids, Petrus J. de Vries, Maylene Shung-King

**Affiliations:** 1 Adolescent Health Research Unit, Division of Child & Adolescent Psychiatry, University of Cape Town, 46 Sawkins Road, Rondebosch, Cape Town, 7700 South Africa; 2 Health Policy and Systems Division, University of Cape Town, Cape Town, South Africa

## Correction to: Child Adolesc Psychiatry Ment Health (2018) 12:6 10.1186/s13034-017-0212-1

After publication of the article [1], it has been brought to our attention that the authors listed in Table 1 are in the wrong order. They should be listed as follows:

- Kashala (2005) [25]
- Cluver (2006) [26]
- Kashala (2006) [27]
- Cluver (2007) [28]
- Menon (2007) [29]
- Okello (2007) [30]
- Cluver (2008) [31]
- Cluver (2009) [77]
- Doku (2009) [34]
- Elhamid (2009) [32]
- Menon (2009) [35]
- Bakare (2010) [36]
- Doku (2010) [37]
- Mueller (2011) [38]
- Puffer (2012) [39]
- Abbo (2013) [40]
- Atilola (2013) [41]
- Cortina (2013) [42]
- Kinyanda (2013) [43]
- Marais (2013) [44]
- Bhana (2014) [45]
- Devries (2014) [46]
- Escueta (2014) [47]
- Lachman (2014) [48]
- Marais (2014) [49]
- Skeen (2014) [51]
- Sharp (2014) [50]
- Waller (2014) [52]
- Asante (2015) [53]
- Casale (2015) [33]
- Chirwa-Mwanza (2015) [62]
- Collishaw (2015) [54]
- Hermenau (2015) [55]
- Mazzucato (2015) [56]
- Okewole (2015) [78]
- Pappin (2015) [57]
- Profe (2015) [58]
- Abdulmalik (2016) [59]
- Bella-Awusah (2016) [60]
- Bhana (2016) [61]
- Cortina (2016) [63]
- Dow (2016) [64]
- Doku (2016) [65]
- Hecker (2016) [66]
- Hensels (2016) [67]
- Lentoor (2016) [68]
- Levetan (2016) [69]
- Okewole (2016) [71]
- Puffer (2016) [72]
- Skeen (2016) [73]
- Sherr (2016) [74]
- Thumann (2016) [75]
- Tucker (2016) [76]
- Mazzucato (2017) [70]

This has now been corrected in the original article.
